# Clinical and radiographic evaluation of low-speed platelet-rich fibrin (PRF) for the treatment of intra-osseous defects of stage-III periodontitis patients: a randomized controlled clinical trial

**DOI:** 10.1007/s00784-022-04627-2

**Published:** 2022-07-25

**Authors:** Yasser Ali Abdulrahman, Manal Mohamed Hosny, Ahmed Elfana, Karim Mohamed Fawzy El-Sayed

**Affiliations:** 1 Department of Oral Medicine and Periodontology, Faculty of Dentistry, Cairo University, Al Saraya Str. 11, Manial, Cairo, Egypt; 2 European Campus Rottal-Inn, Deggendorf Institute of Technology, Deggendorf, Germany; 3 Clinic for Conservative Dentistry and Periodontology, School of Dental Medicine, Christian Albrechts University, Kiel, Germany; 4 Stem Cells and Tissue Engineering Research Group, Faculty of Dentistry, Cairo University, Cairo, Egypt

**Keywords:** Periodontitis, Platelet-rich fibrin, Periodontal regeneration, Intra-bony defects, Randomized controlled trial

## Abstract

**Aim:**

The current randomized controlled trial assessed for the first time the effect of a low-speed platelet-rich fibrin (PRF) with open flap debridement (OFD) versus OFD alone in the treatment of periodontal intra-osseous defects of stage-III periodontitis patients.

**Methods:**

Twenty-two periodontitis patients with ≥ 6 mm probing depth (PD) and ≥ 3 mm intra-osseous defects were randomized into test (PRF + OFD; *n* = 11) and control (OFD; *n* = 11) groups. Clinical attachment level (CAL)–gain (primary outcome), PD-reduction, gingival recession depth (GRD), full-mouth bleeding scores (FMBS), full-mouth plaque scores (FMPS), radiographic linear defect depth (RLDD), and radiographic bone fill (secondary-outcomes) were examined over 9 months post-surgically.

**Results:**

Low-speed PRF + OFD and OFD demonstrated significant intra-group CAL-gain and PD- and RLDD-reduction at 3, 6, and 9 months (*p* < 0.01). Low-speed PRF + OFD exhibited a significant CAL-gain of 3.36 ± 1.12 mm at 6 months (2.36 ± 0.81 mm for the control group; *p* < 0.05), and a significantly greater PD-reduction of 3.36 ± 1.12 mm at 3 months, of 3.64 ± 1.12 mm at 6 months and of 3.73 ± 1.19 mm at 9 months (2.00 ± 0.89 mm, 2.09 ± 1.04 mm, and 2.18 ± 1.17 mm in the control group respectively; *p* < 0.05). No significant differences were notable regarding GRD, FMPS, FMBS, RLDD, or bone fill between both groups (*p* > 0.05).

**Conclusions:**

Within the current clinical trial’s limitations, the use of low-speed PRF in conjunction with OFD improved CAL and PD post-surgically, and could provide a cost-effective modality to augment surgical periodontal therapy of intra-osseous defects of stage-III periodontitis patients.

**Clinical relevance:**

Low-speed PRF could provide a cost-effective modality to improve clinical attachment gain and periodontal probing depth reduction with open flap debridement approaches.

**Supplementary Information:**

The online version contains supplementary material available at 10.1007/s00784-022-04627-2.

## Introduction

Periodontitis is a multifactorial inflammatory progressive destructive disorder of the periodontium, associated with microbial dysbiosis, which untreated can lead to teeth loss and systemic effects [1–7]. Although functional periodontal regeneration remains to be the ultimate goal for periodontal therapy, this endeavor is challenged by the biological intricacy of the periodontal support with the soft tissue components (periodontal ligament and gingiva) integrated and connected complexly into its hard tissues (alveolar bone and cementum) [8–10]. In this context, a variety of techniques and materials have been suggested to achieve a complete healing/regeneration of lost periodontal support [8, 11, 12].

Platelet-rich fibrin (PRF), the second generation of platelet concentrates, has been introduced as an autologous biological scaffold for periodontal therapy [13–15]. It harbors a wide variety of biological mediators integrated in its fibrin matrix, including the platelet-derived growth factor (PDGF), the vascular endothelial growth factor (VEGF), the transforming growth factor beta (TGF-β), and the insulin-like growth factor (IGF) [16–18], which are slowly released over its degradation time [19]. Clinically, PRF matrices proved to enhance periodontal regeneration. In a systematic review investigating the adjunctive effects of platelet-rich plasma (PRP), PRF, enamel matrix derivative (EMD), and amnion membrane (AM) combined with bone grafts on the treatment of intra-osseous periodontal defects, PRF has shown to be the most effective regenerative adjunct [20]. Additionally, PRF application in conjunction with open flap debridement (OFD) procedures yielded greater periodontal regeneration compared to OFD alone or to PRF in combination with bone grafting materials [14, 21].

As a development of the original PRF protocol, a low-speed centrifugation concept was introduced to increase the platelets, leucocytes, and growth factors contained within the PRF matrix [22–24], resulting in a superior in vitro growth factors release profile compared to earlier PRF preparation protocols [25] and increased migration and proliferation of fibroblasts during wound healing [26]. The present randomized controlled trial aimed to compare for the first time the clinical attachment level gain (CAL-gain; primary outcome), probing depth (PD-reduction), gingival recession depth (GRD), full-mouth bleeding (FMBS) and plaque scores (FMPS), radiographic linear defect depth (RLDD), and radiographic bone fill (secondary outcomes) of a low-speed PRF with open flap debridement (PRF + OFD) versus OFD alone for the treatment of intra-osseous periodontal defects of stage-III periodontitis patients.

## Materials and methods

### Study design

The present study was conducted as a parallel-group randomized controlled trial with 1:1 allocation ratio to investigate the clinical and radiographic effects of low-speed PRF utilized with OFD (test group) compared to OFD alone (control group) for the treatment of periodontal intra-osseous defects. The trial protocol was registered at www.clinicaltrials.gov on April 2019 (NCT03924336). The research ethics committee of the Faculty of Dentistry, Cairo University in Egypt approved the trial’s protocol on May 2019 (IRB:19–5-2). The trial was conducted in accordance with the EQUATOR guidelines and the ethical principles of the Helsinki declaration as revised in Fortaleza in 2013, and reported as recommended by CONSORT statements.

### Study population

Participants’ recruitment, operation, and follow-up were carried out between September 2019 and May 2021 at the Department of Periodontology, Faculty of Dentistry, Cairo University, Egypt. Included participants were 18 years in age or older, did not have any previous surgical periodontal therapy at the treatment site, diagnosed with stage-III periodontitis with CAL ≥ 5 mm, PD ≥ 6 mm, which persisted 6–8 weeks following non-surgical periodontal therapy [12], with two or three-walled intra-osseous defect, and FMBS [27] and FMPS [28] less than 20% [29, 30]. Exclusion criteria were the presence of tooth mobility, intra-osseous defects extending to the furcation area, smokers, presence of systemic condition that could affect periodontal healing (e.g., diabetes or hyperthyroidism), history of radiotherapy, chemotherapy or bisphosphonate intake, active orthodontic therapy, and pregnant or lactating females [30, 31].

### Sample size

The sample size was calculated for CAL-gain as the primary outcome. Effect size was derived from a previous study [32], which demonstrated CAL-gain in PRF + OFD of 3.65 ± 1.09 mm, while the OFD-only group showed a mean of 2.31 ± 0.73 mm at 9 months post-operatively. Using a two-sided *t*-test and with type I error set at 0.05 and power of 80%, 9 participants were deemed necessary in each group, which were increased by 20% to 11 participants per group to account for possible dropouts during the trial’s period. Calculations were performed, using G-Power software Version 3.1 (Heinrich-Heine-Universität, Düsseldorf, Germany).

### Randomization and blinding

A random sequence was generated, with 1:1 allocation ratio (www.random.org), and concealed in serial, identical opaque sealed envelopes (MH). All cases were equally prepared for the surgical day and operated by a single periodontist (YA). Allocating participants in either test (PRF + OFD) or control (OFD) groups was carried out on the day of surgery after flap reflection by the study coordinator (KFE). Due to the nature of study interventions, neither participants nor investigator could be blinded. Outcome assessors and statistician were blinded to the participants’ identities and their allocated intervention group.

### Interventions

#### Pre-operative phase

**Fig. 1:**
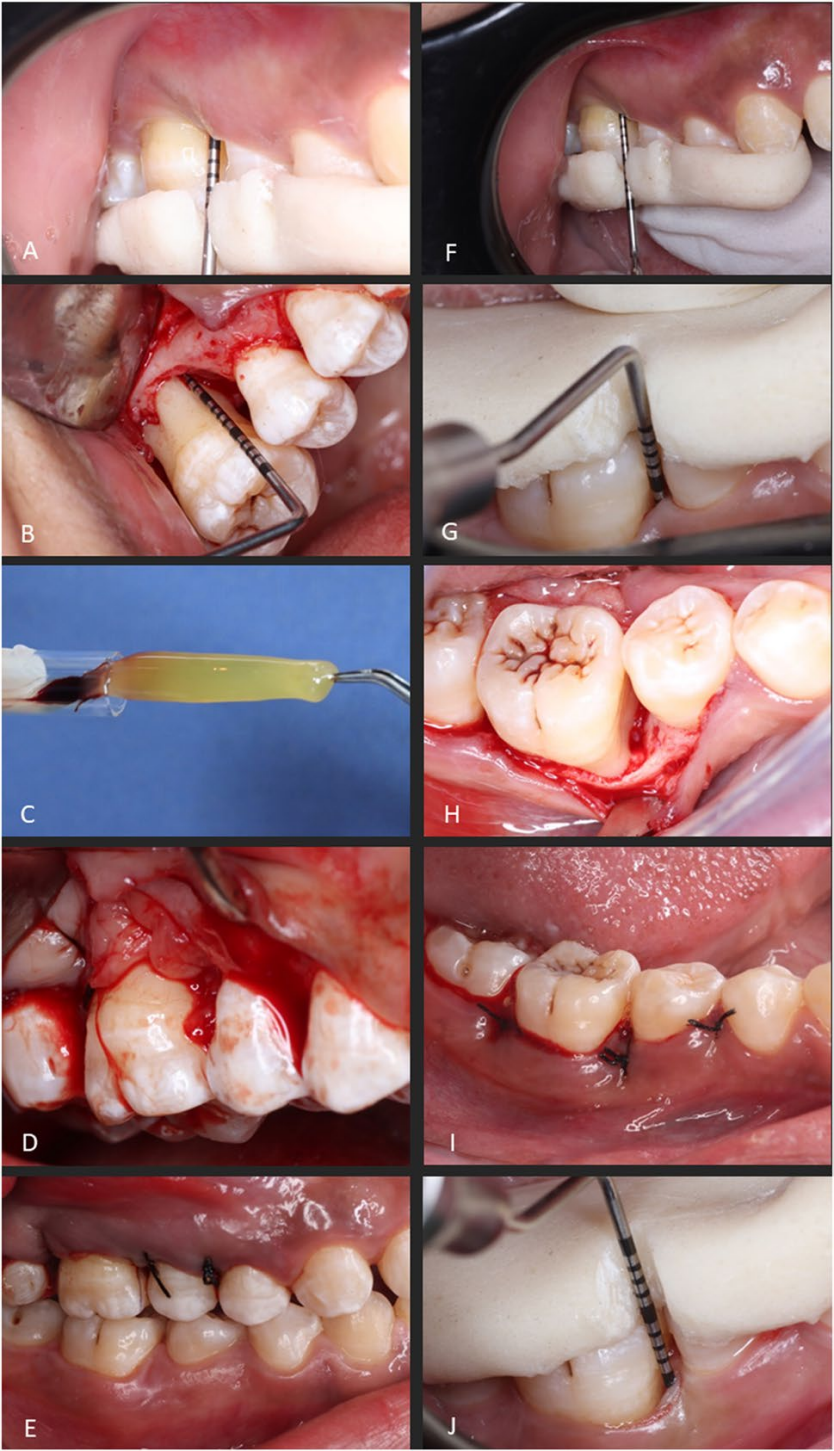
Clinical steps for test (**A**–**F**) and control (**G**–**J**) groups. Test group: baseline measurements taken using a prefabricated stent (**A**), intra-osseous defect at the mesial site of upper right first molar (**B**), low-speed platelet-rich fibrin (PRF) preparation (**C**), low-speed PRF placed into the intra-osseous defect (**D**), flap approximation and suturing (**E**), and final clinical measurements 9 months post-operatively (**F**). Control group: baseline measurements taken using a prefabricated stent (**G**), intra-osseous defect at the mesial site of lower first molar (**H**), flap approximation and suturing (**I**), and final clinical measurements 9 months post-operatively (**J**)

For participants initially fulfilling the inclusion criteria, the study’s details and timeline were explained. The participants signed informed consents, proceeded to the non-surgical therapy phase comprising of supragingival scaling and subgingival debridement, and were instructed in oral hygiene performance. After 6 to 8 weeks, re-evaluation was carried out to confirm the necessity for surgical periodontal therapy. Custom-made acrylic stents with interproximal guiding grooves were fabricated to standardize clinical measurements throughout the study’s duration (Fig. 1). Additionally, bite registration blocks were constructed to guide the film holder to standardize the periapical radiographs, using the paralleling-technique.

#### Surgical interventions

All surgical procedures were performed by a single periodontist (YA). After the administration of local anesthesia (2% mepivacaine hydrochloride levonordefrin 0.005%, Alexandria Co. for Pharmaceuticals, Alexandria, Egypt), intrasulcular incisions were made, using a 15c blade (Trinon Titanium GmbH, Karlsruhe, Germany) then full-thickness mucoperiosteal flaps were raised buccally and lingually. Debridement of the surgical site was performed using ultrasonic scalers (Woodpecker Medical Instrument Co., Guilin, China) and Gracey curettes (Miltex, Hessen, Germany). Confirmation of the surgical defect morphology was visually carried out and the allocation sequence was revealed. For the test group (PRF + OFD), the low-speed PRF was prepared through collecting 10 mL of fresh blood via venipuncture of the forearm into a sterile glass vacuum tube (16 × 100 mm, 10 mL, Voma Med, Chongqing, China) and processed, based on previously reported protocols for low-speed and advanced PRF (A-PRF +) preparation [23, 25], using a digital tabletop centrifuge (VE-4000, Velab, TX, USA) with a rotor angle of 45° and a maximum radius of 10.6 cm operated at 1300 RPM (maximum relative centrifugal force (RCF-max) = 200 g) for 8 min at room temperature. The obtained PRF was compressed using a sterile gauze and inserted into the periodontal defect. For the control group (OFD alone), no biomaterial was used. Flaps were approximated in both groups with interrupted 4–0 silk sutures to achieve primary closure (Hu-Friedy, IL, USA; Fig. 1).

#### Post-operative phase

Participants were prescribed 875 mg amoxicillin + 125 mg clavulanate (Augmentin 1 g, GlaxosmithKline, Worthing, England) post-operatively twice daily for 7 days in addition to ibuprofen 600 mg (Brufen, Kahira Pharma Co., Cairo, Egypt) three times daily for 3 days. Moreover, participants were instructed to avoid tooth brushing and trauma to the surgical site and to rinse twice daily with 0.12% chlorhexidine HCl (Hexitol, ADCO Pharma, Egypt) for 2 weeks [33]. Sutures were removed 2 weeks post-operatively and participants were advised to resume mechanical biofilm removal using soft tooth brushes. Participants were recalled weekly at the first month then at 3, 6, and 9 months post-operatively to monitor surgical site healing and proper oral hygiene practices and assess the study’s outcomes.

#### Outcomes

Clinical attachment level (CAL) was measured, using a UNC-15 periodontal probe and a pre-fabricated custom stent, as the distance from the base of the periodontal pocket to the cemento-enamel junction (CEJ) in millimeters, and CAL-gain (primary outcome) was calculated by subtracting follow-up CAL values from baseline values. Probing depth (PD) was measured as the distance from the base of the pocket to the gingival margin, and the gingival recession depth (GRD) was determined as the level from the gingival margin to the CEJ at baseline and 3, 6, and 9 months post-operatively. Similarly, changes in these outcomes were calculated by deducting values of 3, 6, and 9 months from baseline, and change percentages were calculated as a proportion from baseline values. Full-mouth bleeding scores (FMBS) and full-mouth plaque scores (FMPS) were determined at baseline and 9 months.

For radiographic analysis, standardized periapical radiographs were taken, using long-cone paralleling method with E-speed films (YES!Star, Zhengzhou Smile Dental Equipment Co., Ltd, Zhengzhou, China) mounted in a custom-made bite block and a holder kit (XCP film holder set, Dentsply Sirona, PA, USA). The x-ray machine (Heliodent Plus, Dentsply Sirona, PA, USA) was set at standardized exposure parameters (60 kVp, 8 mA, 0.7 mm focal spot, and 0.3 s exposure time). Films were scanned (Xios Scan, Dentsply Sirona, PA, USA) and transferred into an image processing software (Planmeca Romexis, V.6, all-in-one dental imaging software, Helsinki, Finland). The baseline radiographic defect angle was measured as the angle formed by the bony wall of the defect with the long axis of the tooth, and the linear radiographic defect depth (RLDD) was determined from the base of radiographic bone defect to the alveolar crest, which was measured as reported previously [30, 34] at baseline and 6 and 9 months. Radiographic bone fill in millimeters was calculated by subtracting follow-up values from baseline, and percentages were expressed as proportion of bone fill of the baseline RLDD.

#### Calibration

An experienced periodontist (AE) and an experienced radiologist (MN) not aware of the participants’ corresponding groups obtained all clinical and radiographic measurements respectively throughout the study. Intra-examiner calibration took place before the start of the study by comparing two measurements of the same participants not involved in the study within a 1-week interval, yielding intra-class correlation scores of 0.85 for clinical outcomes and 0.82 for radiographic measurements.

#### Statistical analysis

Categorical data were reported as number (*n*) and percentage (%) and tested for differences using the chi-square test. Numerical data were described as mean ± standard deviation (SD). To explore normality, the Shapiro–Wilk test was used. For normally distributed data, inter-group comparisons took place using an independent *t*-test while intra-group comparisons between different time points were done using repeated-measure ANOVA with Bonferroni adjustment. For non-normally distributed data, the Mann–Whitney test was used for inter-group comparisons and the Friedman test for intra-group comparisons. A stepwise linear regression model was constructed for primary outcome (CAL-gain after 9 months) as the dependent variable, while study group, age, tooth distribution, number of defect walls, baseline radiographic angle, FMBS, and FMBS at baseline and 9 months as well as radiographic bone fill at 9 months were the independent variables. All comparisons were two-tailed and *p* < 0.05 was described as statistically significant. Analyses were conducted using the SPSS software for Windows (version 26, IBM, NY, USA).

## Results

### Participants’ characteristics

The present randomized controlled trial included a total of 22 participants diagnosed with stage-III periodontitis with 22 intra-osseous defects randomized into a low-speed PRF + OFD group (*n* = 11, test group) or an OFD alone group (*n* = 11, control group). The trial was concluded without loss to follow-up as shown in the participants’ flow chart (Figure S1). Healing was uneventful and no unexpected adverse events were reported by participants nor observed clinically (e.g., infection, prolonged bleeding, or surgical site exposure). The test group included 3 male and 8 female participants with a mean age of 35.64 ± 9.59 years and the OFD alone group had 1 male and 10 females with mean age 36.27 ± 9.32 years. Regarding tooth distribution, the test group included 3 anterior, 1 premolar, and 7 molar teeth, while the control group had 4, 3, and 4 teeth respectively. Concerning the intra-osseous defects’ morphology, the test group comprised of 7 two-walled and 4 three-walled defects, while the control group had 9 two-walled and 2 three-walled defects, and the average clinical depths were 3.82 ± 1.08 mm and 3.55 ± 0.82 mm for test and control groups respectively (baseline characteristics are shown in Table 1).

**Table 1:** Participants’ baseline characteristics of age, gender, tooth location, intra-osseous defect morphology, and radiographic defect angle

Baseline characteristics	Low-speed PRF + OFD (*n* = 11)	OFD alone (*n* = 11)	*P*-value
Age (years, mean ± SD)	35.64 ± 9.59	36.27 ± 9.32	0.846
Gender (*n* (%))			
Male	3 (27.3%)	1 (9.1%)	0.269
Female	8 (72.7%)	10 (90.9%)	
Tooth location (*n* (%))			
Anterior	3 (27%)	4 (36%)	0.375
Premolar	1 (9%)	3 (27%)	
Molar	7 (64%)	4 (36%)	
Intra-osseous defect morphology (*n* (%))			
2 walls	7 (64%)	9 (82%)	0.338
3 walls	4 (36%)	2 (18%)	
Intra-osseous defect depth (mm, mean ± SD)	3.82 (1.08)	3.55 (0.82)	0.512
Radiographic defect angle	35.45 ± 8.95	28.64 ± 8.83	0.056

### CAL

A statistically significant gain in CAL was notable between baseline and 3, 6, and 9 months in each group independently (*p* < 0.05). Although CAL-gain was generally higher in the test group, a significant CAL-gain was solely evident at 6 months between test and control groups (3.36 ± 1.12 mm for the test group and 2.36 ± 0.81 mm for the control group, *p* < 0.05; Table 2).

**Table 2:** Clinical outcomes of clinical attachment level (CAL), probing depth (PD), gingival recession depth (GRD), full-mouth bleeding (FMBS), and plaque scores (FMPS)

Clinical outcomes	Low-speed PRF + OFD (*n* = 11)	OFD alone (*n* = 11)	*p*-value
Clinical attachment level (CAL)			
Baseline (mm)	7.91 ± 1.30	7.73 ± 1.56	0.973
At 3 months (mm)	4.73 ± 1.10	5.45 ± 1.51	0.184
At 6 months (mm)	4.55 ± 1.21	5.36 ± 1.43	0.149
At 9 months (mm)	4.36 ± 1.57	5.27 ± 1.49	0.201
Intra-group *p*-value	< 0.001*	< 0.001*	
Gain at 3 months (mm)	3.18 ± 1.25	2.27 ± 0.65	0.053
Gain at 3 months (%)	39.86 ± 12.95	30.30 ± 9.86	0.066
Gain at 6 months (mm)	3.36 ± 1.12	2.36 ± 0.81	0.035*
Gain at 6 months (%)	42.46 ± 12.15	31.21 ± 10.28	0.03*
Gain at 9 months (mm)	3.55 ± 1.37	2.45 ± 0.93	0.05
Gain at 9 months (%)	45.06 ± 17.49	32.35 ± 11.65	0.058
Probing depth (PD)			
Baseline (mm)	7.55 ± 0.93	6.55 ± 1.21	0.057
At 3 months (mm)	4.18 ± 0.60	4.55 ± 1.21	0.522
At 6 months (mm)	3.91 ± 1.08	4.55 ± 1.21	0.249
At 9 months (mm)	3.36 ± 1.12	4.36 ± 1.21	0.305
Intra-group *p*-value	< 0.001*	< 0.001*	
Reduction at 3 months (mm)	3.36 ± 1.12	2.00 ± 0.89	0.006*
Reduction at 3 months (%)	43.72 ± 11.43	30.53 ± 12.81	0.02*
Reduction at 6 months (mm)	3.64 ± 1.12	2.09 ± 1.04	0.003*
Reduction at 6 months (%)	47.80 ± 12.28	31.67 ± 13.99	0.009*
Reduction at 9 months (mm)	3.73 ± 1.19	2.18 ± 1.17	0.01*
Reduction at 9 months (%)	49.10 ± 14.01	32.80 ± 14.98	0.022*
Gingival recession depth (GRD)			
Baseline (mm)	0.55 ± 0.52	1.36 ± 1.36	0.152
At 3 months (mm)	0.73 ± 0.65	1.00 ± 1.00	0.595
At 6 months (mm)	0.73 ± 0.79	1.00 ± 1.00	0.552
At 9 months (mm)	0.64 ± 0.92	1.00 ± 1.00	0.467
Intra-group *p*-value	0.896	0.145	
Full-mouth bleeding score (FMBS)			
Baseline (%)	10.00 ± 2.65	10.36 ± 2.46	0.522
At 9 months (%)	12.91 ± 3.33	15.45 ± 6.61	0.267
Full-mouth plaque score (FMPS)			
Baseline (%)	9.82 ± 2.71	10.36 ± 2.46	0.339
At 9 months (%)	13.36 ± 3.53	15.36 ± 4.32	0.248

^*^Statistical significance is marked with asterisk

### PD

In both groups, there was a statistically significant decrease in PD between baseline and 3, 6, and 9 months (*p* < 0.05). Compared to the control group, the test group showed significantly higher PD-reduction of 3.36 ± 1.12 mm at 3 months, of 3.64 ± 1.12 mm at 6 months, and of 3.73 ± 1.19 mm at 9 months (2.00 ± 0.89 mm, 2.09 ± 1.04 mm, and 2.18 ± 1.17 mm in the control group, respectively, *p* < 0.05; Table 2).

### GRD, FMBS, and FMPS

No intra- or inter-group differences were notable regarding GRD changes, FMBS, or FMPS (*p* > 0.05; Table 2).

### Radiographic analysis

RLDD and radiographic bone fill significantly improved in each of the study groups (*p* < 0.05), with no significant differences observed between the groups (*p* > 0.05; Table 3).

**Table 3:** Radiographic outcomes of radiographic linear defect depth (RLDD) and radiographic bone fill

Radiographic analysis	Low-speed PRF + OFD (*n* = 11)	OFD alone (*n* = 11)	*p*-value
Radiographic linear defect depth (RLDD) (mm)			
At baseline	7.91 ± 1.30	7.73 ± 1.56	0.769
At 6 months	6.29 ± 1.24	6.65 ± 1.22	0.497
At 9 months	5.82 ± 1.19	6.41 ± 1.41	0.300
Intra-group *p*-value	< 0.001*	< 0.001*	
Radiographic bone fill (mm)			
At 6 months	1.62 ± 0.73	1.07 ± 1.07	0.234
At 9 months	2.09 ± 0.73	1.32 ± 1.21	0.156
Radiographic bone fill (%)			
At 6 months	20.39 ± 8.74	12.77 ± 12.98	0.178
At 9 months	26.30 ± 8.35	17.91 ± 12.97	0.130

^*^Statistical significance is marked with asterisk

### Regression analysis

The stepwise linear regression analysis demonstrated that among all independent variables investigated, the radiographic bone fill showed a significantly positive correlation with the CAL-gain after 9 months post-surgically (*p* = 0.04, Table 4).

**Table 4:** Stepwise linear regression analysis for clinical attachment level gain (CAL-gain) after 9 months as the dependent variable

	*β*	SD	95% CI		*p*-value
			Lower limit	Upper limit	
Treatment group	− .643	.601	− 1.965	.679	.307
Age	.019	.047	− .085	.123	.699
Number of walls	− .020	.666	− 1.487	1.447	.977
Tooth distribution	.678	.326	− .040	1.397	.062
Radiographic angle at baseline	− .017	.031	− .084	.051	.598
FMPS at baseline	.234	.661	− 1.221	1.689	.730
FMPS at 9 months	− .038	.103	− .265	.190	.724
FMBS at baseline	− .241	.705	− 1.794	1.311	.739
FMBS at 9 months	.061	.075	− .105	.227	.438
Radiographic bone fill at 9 months	.581	.255	.019	1.143	.044*

*β*, regression coefficient; *SE*, standard error; *CI*, confidence interval; *FMPS*, full-mouth plaque score; *FMBS*, full-mouth bleeding score. *Statistical significance differences are marked with asterisk

## Discussion

Periodontitis, a multifactorial chronic inflammatory disorder of the teeth supporting structures [2], culminates in periodontal tissue destruction with horizontal and vertical osseous defects, commonly accompanied with deep residual pockets, which worsen the affected teeth prognosis [35–37]. Mechanical removal of etiological and contributing factors [38, 39] remains to be the primary step of any periodontal therapy. In this context, OFD remains to be one of the most documented evidence-based approaches for the surgical treatment of intra-osseous defects with remarkable clinical outcomes [37, 40, 41]. Yet, although OFD could enhance clinical and radiographic parameters, histologically it mostly results in healing in the form of “repair,” with long junctional epithelium forming a new attachment over the affected cementum [42]. Still, a restoration of the lost tooth supporting structures remains to be the utmost goal of periodontal therapy, with vertical intra-osseous defects showing greater potential for periodontal regeneration [43, 44].

The aim of the current randomized controlled trial was to assess clinically and radiographically the periodontal healing/regenerative potential of a low-speed PRF delivered into intra-osseous defects through OFD, in comparison to OFD alone over a 9 months observation period. In the present trial, smokers were excluded to avoid the negative effects of smoking on periodontal healing/regeneration [45, 46]. Apart from the heterogeneity in PRF preparation devices and protocols, it has been demonstrated that low-speed PRF of comparable quality can be reproduced successfully irrespective of the commercial centrifugation device when utilizing the same centrifugal speed and force [47]. Thus, the present trial employed previously reported standard speed and force parameters to prepare the low-speed PRF [48, 49]. Despite the advantages of a split-mouth design, including the control for confounders, as each patient would serve as his own control, as well as possible sample size reduction, a parallel design was chosen to eliminate any possibility for a systemic “carry-across” effect, in which local diffusion of the PRF enclosed growth/differentiation factors from intervention sites could influence the healing at the control sites [50]. In the present study, CAL-gain was defined as the primary outcome, being the most universally accepted surrogate parameter for evaluating periodontal healing/regeneration [12] and a direct prognostic factor related to true periodontal “hard” endpoints as tooth-survival [51].

PRF, with its various and continuously evolving preparation protocols, has opened new perspectives to improve clinical outcomes of periodontal therapies over the last years [14, 21]. Compared to conventional PRF, low-speed PRF is reported to demonstrate a significant higher accumulated release of VEGF, TGF-β1, and EGF [22], with a growth/differentiation factors release profile superior to L-PRF or A-PRF [25], favoring fibroblasts’ migration/proliferation during periodontal wound healing [26]. A recent investigation demonstrated a significant healing/regenerative potential for the low-speed A-PRF + comparable to EMD in the treatment of intra-osseous periodontal defects 6 months postoperatively [52]. Combining A-PRF + with an alloplastic mixture, composed of 70% hydroxyapatite and 30% β-tricalcium phosphate, for alveolar bone preservation/augmentation resulted in significantly less post-operative swelling and pain [53]. Previous randomized controlled clinical trials comparing PRF [54], titanium-prepared PRF [32], PRF in combination with 1.2% atorvastatin [55], or A-PRF [56] applied with OFD versus OFD alone demonstrated enhanced periodontal healing with higher PD-reduction, CAL-gain, and radiographic defect fill in the platelets concentrate compared to the OFD groups. Similarly, in the current study, low-speed PRF with OFD significantly improved CAL-gain at 6 months as well as PD-reduction for up to 9 months. The stepwise linear regression analysis further demonstrated a significant correlation between CAL-gain and radiographic bone fill. Apart from the physical characteristics of the defect filling PRF hemostatic plug, the observed beneficial periodontal clinical outcomes can be explained relying on the release of the abovementioned growth, differentiation, and angiogenic factors as well as adhesion and coagulation biomolecules by the low-speed PRF, resulting in favorable cellular and biological effects, comprising the induction of a heightened migration and proliferation of gingival and periodontal fibroblasts [26], as well as their increase in expression of collagen type 1, PDGF, and TGF-β [25]. Finally, through its fibrin content, the low-speed PRF plug would represent an essential three-dimensional scaffold/framework for the resident periodontal cells, enhancing their local micro-environment during the biological healing/regeneration events.

Still, the results of the present randomized controlled clinical trial should be carefully interpreted in context of its limitations. First, the preparation of blood-derived biomaterials such as PRF requires collection of the patient’s own blood. Consequently, patients who were anxious of this procedure refused to participate in the present study. Second, blinding of participants could not be implemented due to the nature of procedure as the test group required blood sample collection. Third, although a 9-month follow-up period may be an acceptable period for evaluating healing and bone remodeling in periodontal defect, longer follow-up periods remain to be desirable to evaluate true periodontal endpoints (e.g., tooth survival). Yet, this was not feasible with the current study’s population from lower socio-economic background, visiting the Faculty of Dentistry, Cairo University, primarily for symptomatic treatment and considering repeated visits over a longer period a burden to their daily life. Fourth, despite the fact that in the current investigation a conventional UNC-15 periodontal probe was used for recording the periodontal findings, being a cost-effective modality of acceptable accuracy in the hands of a calibrated operator, the use of pressure sensitive periodontal probes could have additionally heightened the sensitivity and accuracy of the recorded surrogate parameters. Fifth, the current study did not record patient-related outcomes (e.g., postoperative pain, swelling, bleeding, outcomes related to the venipuncture). Finally, as in most clinical trials, the true nature of the achieved periodontal healing/regeneration could not be verified through a histological analysis for evident ethical reasons, but had to be indirectly assumed through surrogate clinical and radiographic parameters.

Within the limitation of the present randomized controlled clinical trial, it can be concluded that both OFD alone or in conjunction with low-speed PRF were able to produce significant improvement in clinical (CAL-gain and PD-reduction) and radiographic parameters (RLDD) in the treatment of periodontal defects 9 months post-surgically. The presence of low-speed PRF in the test group resulted in superior CAL-gain and PD-reduction and hence can be considered a viable cost-effective addition for improving periodontal healing/regeneration with OFD. Future research is required to explore possible advancements in blood collection tube compositions and their influence on the obtained low-speed PRF volume and quality [32, 47]. Horizontal centrifugal procedures, which are postulated to enhance PRF inclusion and uniform distribution of platelets and leucocyte [57, 58], should be further investigated with various centrifugal speed and force settings, with special emphasis on optimization of the regenerative and antibiotics/biological delivery potential of low-speed PRF (30, 59). Finally, further studies with longer follow-up periods are needed to confirm the reported effects, especially in comparison to different PRF preparation schemes (e.g., L-PRF) or in combination with periodontal biomaterials (bone grafts or biological agents).

## Supplementary Information

Below is the link to the electronic supplementary material.

Supplementary file1 (DOC 218 KB)

Supplementary file2 (DOC 50 KB)
