# Supplementary material for: Clinical and radiographic evaluation of low-speed platelet-rich fibrin (PRF) for the treatment of intra-osseous defects of stage-III periodontitis patients: a randomized controlled clinical trial
Source: Clin Oral Investig. 2022 Jul 25;26(11):6671–80. doi: 10.1007/s00784-022-04627-2 (PMC9643252; doi:10.1007/s00784-022-04627-2)
Supplement: Supplementary file 2 — Supplementary file2 (DOC 50 KB) [file 784_2022_4627_MOESM2_ESM.doc]

**
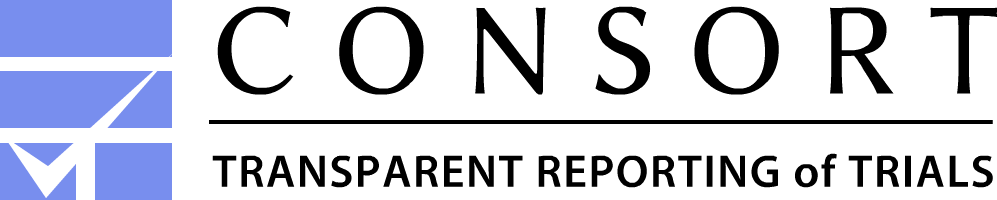
**

**CONSORT 2010 Flow Diagram**

**Allocation**

**Analysis**

**Follow-Up**

**Enrollment**

Assessed for eligibility (n=45 )

Excluded (n= 23)

♦  Not meeting inclusion criteria (n= 12)

♦  Declined to participate (n= 11)

♦  Other reasons (n= 0)

Analysed (n= 11)
♦ Excluded from analysis (n= 0)

Lost to follow-up (n= 0)

Discontinued intervention (n= 0)

Allocated to A-PRF+ with OFD (test) (n= 11)

♦ Received allocated intervention (n= 11)

♦ Did not receive allocated intervention (n= 0)

Lost to follow-up (n= 0)

Discontinued intervention (n= 0)

Allocated to OFD only (control) (n= 11)

♦ Received allocated intervention (n= 11)

♦ Did not receive allocated intervention (n= 0)

Analysed (n= 11)
♦ Excluded from analysis (n= 0)

Randomized (n= 22)
